# The Correlation Between Effort–Reward Imbalance at Work and the Risk of Burnout Among Nursing Staff Working in an Emergency Department—A Pilot Study

**DOI:** 10.3390/healthcare12222249

**Published:** 2024-11-11

**Authors:** Justus Wolfgang Braun, Sabine Darius, Irina Böckelmann

**Affiliations:** Department of Occupational Medicine, Otto-von-Guericke-University Magdeburg, 39120 Magdeburg, Germany; sabine.darius@med.ovgu.de (S.D.); irina.boeckelmann@med.ovgu.de (I.B.)

**Keywords:** psychological stress, nursing, emergency medicine, effort–reward imbalance, burnout

## Abstract

**Background**: Nurses in emergency rooms face high levels of psychological stress during their work, which is why they have an increased risk of burnout. The aim of this pilot study was to determine the extent to which effort–reward imbalance (ERI) at work is connected to the risk of burnout among emergency nurses. **Methodology**: Thirty-five nurses from the central emergency department of a maximum care provider, aged 35.1 ± 0.6 years, voluntarily participated in this pilot study. ERI was recorded using the Effort–Reward Imbalance questionnaire. The nurses were then divided into two groups: the nonrisk group (ERI ratio < 1, *n* = 19) and the risk group (ERI ratio ≥ 1, *n* = 16). The burnout dimensions were determined via the Maslach Burnout Inventory (MBI) and the burnout risk was then determined. **Results**: Nurses in the nonrisk group were significantly less emotionally exhausted (1.6 ± 1.1 points) and had a lower overall risk of burnout (MBI total score 1.2 ± 0.67 points) than the nurses of the risk group. (3.3 ± 1.5 and 2.2 ± 1.2 points, respectively). Eighteen nurses from the total sample had a medium or high risk of burnout. There is a correlation between the risk of burnout and the ERI ratio (r = 0.392, *p* < 0.05) and the ERI scale “job promotion” (r = −0.462, *p* < 0.05). **Conclusions**: The pilot study provides indications of a relationship between effort–reward imbalance at work and burnout risk in emergency nurses. These findings may improve rewards for nurses in the form of increased salary and/or recognition to reduce the risk of burnout among employees and avoid turnover. Further research is needed to investigate the influence of preventive measures on the risk of burnout and gratification crises, particularly with regard to the influence of status and recognition on the risk of burnout.

## 1. Introduction

Employees working in emergency departments face high levels of stress during their work [1]. Various international studies have reported an increased risk of burnout among nurses working in this area [2]. Many reasons may explain the increased psychological stress observed among nursing staff working in the field of emergency care. According to the concept of stress–strain developed by Rohmert und Rutenfranz [3], mental strain is a consequence of stress, which is understood in terms of the totality of the various external influences that affect the individual. Depending on the type of stress and the individual conditions of the person affected, such strain can lead to short-term stress reactions or long-term health impairments. Depending on the working environment and individual factors, stress (e.g., due to high patient volume) can lead to both positive reactions (e.g., increased alertness) and negative stress response (exhaustion, fatigue). Chronic negative stress, defined as long-term stress that goes on for weeks or months, can lead to the development of the burnout syndrome. The term “burnout” was originally used in the psychological sense in 1974 by the psychoanalyst Herbert Freudenberger, who classified it as a reduction in one’s own strength and performance due to previous overload. He was referring in particular to the group of people working in a voluntary capacity [4]. Maslach and Jackson made further concrete attempts to define burnout. They classified burnout based on three aspects: emotional exhaustion, depersonalization and reduced personal accomplishment. They saw burnout syndrome primarily as a disease of employees in social professions [5]. Since then, the definition of burnout syndrome based on the three dimensions has been scientifically debated [6] but is still the common systematic approach [7]. Today, burnout is defined as “a syndrome conceptualized as resulting from chronic workplace stress that has not been successfully managed” [7].

Chronic mental stress (including burnout, defined as long-term stress that goes on for weeks or months), is highly relevant with regard to the well-being of employees and leads to higher rates of absenteeism as well as cardiovascular diseases and depression [8]. In addition to high professional demands, other factors can lead to a high risk of burnout, including low levels of reward and limited latitude regarding job decisions [9,10].

High work demands (stress in the sense of the stress–strain concept) on the one hand and low rewards (strain in the sense of this concept) on the other can lead to health impairments. The purpose of the effort–reward imbalance model (ERI; Ref. [11]) is to identify the stress experienced at work and the associated health effects on the employee. Occupational exertion includes factors such as time pressure, responsibility and overtime to be worked, which affect the employee in the workplace. In addition to income, the employee’s rewards also include job security, recognition and appreciation as well as possible career opportunities. According to Siegrist, an effort–reward imbalance can occur among employees if, over a long period of time, the effort they invest is mismatched with their perceived rewards, which can take the form of recognition, promotion opportunities or job security [11]. A lack of reciprocity between the effort invested and the reward received represents a chronic stress experience. Various studies have demonstrated links between effort–reward imbalance at work and cardiovascular events, gastrointestinal and musculoskeletal symptoms and mental illnesses [11,12,13,14,15,16]. A correlation between emotional exhaustion or reduced personal accomplishment and the experience of effort–reward imbalance at work has also been observed among nurses working at a university hospital, especially among nurses who invest a great deal of intrinsic effort into their work [9]. A correlation between emotional exhaustion or reduced personal accomplishment and the experience of effort–reward imbalance at work has also been observed among nurses working at a university hospital, especially among nurses who invest a great deal of intrinsic effort into their work [12]. The connection between effort–reward imbalance and burnout can be explained by the impact on the perception of work through the individual in case of a high-effort and low-reward situation. High effort and low rewards can lead to a negative perception of their own work and associated feelings of frustration and negative emotions, which increase the risk of burnout. A link between effort–reward imbalance and burnout has also been observed among emergency room physicians [17]. However, few studies have analyzed the frequency of effort–reward imbalance at work among nursing staff working in the field of emergency care. Previous studies on this topic have been conducted outside Germany and have indicated an increased risk of effort–reward imbalance at work among emergency care workers [18,19].

As these studies are lacking in Germany, it is important to close this gap. Due to the prevailing shortage of skilled workers in Germany, which is also serious in the care sector, the health of staff should be maintained. In addition, the workload in the emergency department has increased during the COVID-19 pandemic [20,21]. To this end, it is necessary to minimize the risk of burnout. A precise determination of the level of effort–reward imbalance at work exhibited by nursing staff working in German emergency departments and the associated risk of burnout may contribute to mitigate the expected shortage of skilled nurses both in general [22] and in the specific context of emergency services [23]. The aim of this pilot study was to determine the extent to which effort–reward imbalance at work is connected to the risk of burnout among emergency nurses during COVID-19-pandemic. The study therefore examined the following questions:

1. How many emergency nursing staff members face an increased risk of effort–reward imbalance at work?
2. How high is the risk of burnout among this occupational group?
3. Is the risk of burnout correlated with effort–reward imbalance at work among emergency nurses?
4. Which components of reward (esteem, job promotion, job security) are related to the risk of burnout?

Answering these questions will assist in establishing preventive measures aimed at maintaining the health of emergency nursing staff, ensuring that nursing staff remain in the profession and counteracting the issues of staff turnover and skills shortages.

## 2. Materials and Methods

### 2.1. Sample and Recruitment

All nurses in the central emergency department (CED) were asked to take part in the survey. All volunteers who had worked in the emergency department for more than one year were included. Medical staff were excluded, as were nurses with less than one year’s experience and trainees. All participants were given a questionnaire. Thirty-seven nursing staff members working in the CED of a maximum care hospital voluntarily participated in this study. Two participants did not complete the questionnaire in full. In the end, the sample consisted of 35 nurses aged 19–65 years (mean 35.2 ± 10.81 years). Subjects were recruited from August 2021 to January 2022 during the COVID-19 pandemic via interviews, at which the subjects were informed of the objectives and procedure of this study. The response rate was 90%.

### 2.2. Questionnaire

First, sociodemographic and job-related data were collected. Effort–reward imbalance was assessed via the German version of the Effort–Reward Imbalance Questionnaire (ERI) developed by Siegrist et al. [24], and the risk of burnout was assessed via the German version of the Maslach Burnout Inventory-General Survey (MBI-GS) [25].

#### 2.2.1. Effort–Reward Imbalance Questionnaire

This questionnaire, which was drawn from Siegrist et al. [24], examines professional “effort” and “reward”. A short version of the questionnaire featuring 10 items was used in this research (3 items were used to measure effort, while 7 items were used to measure reward) [24]. “Reward” is divided into the subscales “job promotion”, “esteem” and “job security”. Responses were scored on a four-point Likert scale ranging from “strongly disagree” to “strongly agree”. The ratio of expenditure to reward (ERI ratio) was then calculated [24], in which context an ERI ratio < 1 was rated as “reward sufficient considering the effort”, while an ERI ratio ≥ 1 was rated as “reward insufficient considering the effort”. A high ERI ratio indicates a high degree of perceived imbalance between effort and reward; a value ≥ 1 is considered to be hazardous to the individual’s health. This value was used to divide the overall sample into a risk group (ERI ≥ 1) and a nonrisk group (ERI < 1). Cronbach’s α for the subscale effort is 0.854, and for reward 0.835 (in both cases, the level of reliability is very reliable).

#### 2.2.2. Maslach Burnout Inventory

The German version of the standardized MBI-General Survey (MBI-GS) [26] developed by Büssing and Perrar [27] was used as a diagnostic instrument to assess burnout symptoms and to determine the extent of occupational burnout. This questionnaire is based on the MBI, which was developed by Maslach and Jackson [5]. The risk of burnout was determined via the MBI General Survey developed by Maslach et al. [26]. The three dimensions of “emotional exhaustion”, “depersonalization” and “personal accomplishment” are measured. The respondents answered questions on a seven-point scale ranging from “never true” to “true every day”. Based on the average frequency of symptoms, the levels of the three MBI dimensions were classified into the subcategories “low”, “average” and “high”. Individual burnout risk was calculated in accordance with the suggestions of Kalimo [28]. All three weighted MBI dimensions are included. The test subjects were then divided into the following groups according to the total MBI score: “no burnout”, “some symptoms” and “burnout”. Cronbach’s α for the subscale emotional exhaustion is 0.914; for depersonalization, 0.816 (in both cases the level of reliability is very reliable); and for personal accomplishment, 0.755 (reliable). 

### 2.3. Statistical Analysis

Statistical analysis was performed via SPSS version 29 software (IBM, Armonk, NY, USA). The significance level was set at *p* < 0.05. The mean values, standard deviations, medians, minimum and maximum values, and 95% confidence intervals were calculated descriptively. The normality of the data was tested via the Shapiro—Wilk test. Contingency tables, the chi-square test or Fisher’s exact test, and the Mann—Whitney U test for two independent samples (ERI groups) were used to support the statistical group comparison analyses. Statistical relationships were calculated via Spearman’s correlation analysis; for the interpretation of the results, the interpretation of Cohen [29] was used.

### 2.4. Ethics

This study was conducted in compliance with the Declaration of Helsinki and the European General Data Protection Regulation (GDPR2016/679). Permission was sought and obtained from the Ethics Committee of the Otto-von-Guericke University at Medical Faculty (Reg.-Nr. 97/21). Before data collection, the participants were informed of the study’s aims and the possibility of terminating their participation in the study without suffering any consequences. Written informed consent was obtained from each participant.

## 3. Results

On the basis of the results of the ERI questionnaire, 16 (45.7%) subjects were assigned to the risk group (i.e., those with an ERI score ≥ 1), while 19 (54.3%) subjects were assigned to the nonrisk group (i.e., those with an ERI score < 1). No significant differences were observed between the two ERI groups in terms of age, sex or years of work experience in the CED (Table 1).

The values of the subscales of the reward scale were determined on the basis of the ERI questionnaire and compared between the two ERI groups (Table 2).

The degrees of severity of the burnout dimensions for the total sample and the two ERI groups are presented in Table 3. The distribution of the degrees of severity between the two groups examined was statistically significant only with respect to “emotional exhaustion” (*p* < 0.05). A total of 5.3% of the subjects in the nonrisk group and 56.3% of those in the risk group exhibited high levels of “emotional exhaustion”. Moreover, 25% of the nursing staff with ERI ≥ 1 were at risk of burnout, and 50% exhibited some burnout symptoms. In the nonrisk group, the corresponding figures are 0 and 31.6% of the subjects, respectively (*p* < 0.05).

**Figure 1:**
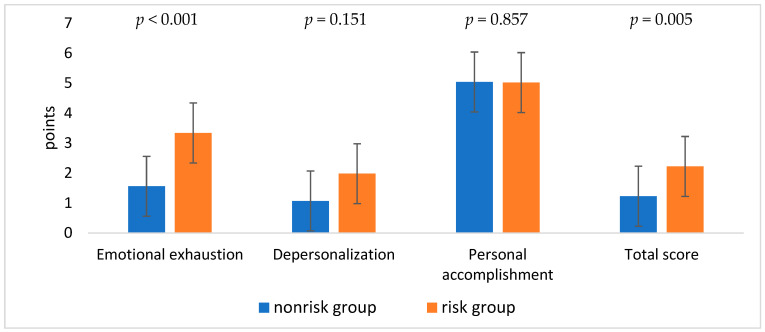
Characteristics of the burnout dimensions between the two ERI groups.

The comparison between the two ERI groups revealed higher values for “emotional exhaustion” and the total MBI score among the risk group (*p* < 0.05; Figure 1).

Spearman correlation analyses were performed to identify the relationships between burnout and ERI (Table 4). “Emotional exhaustion” and “depersonalization” are negatively correlated with reward and status. “Personal accomplishment” is not related to any ERI scale or subscale.

The overall risk of burnout is positively correlated with effort and the risk of an effort–reward imbalance at work and negatively correlated with reward and financial reward. The greater the effort and perceived effort–reward imbalance at work are, the greater the emotional exhaustion expressed by the respondents.

## 4. Discussion

In this pilot study, 47% of the nursing staff exhibited an increased risk of effort–reward imbalance at work. Overall, an average ERI ratio of 1.00 was determined across all respondents. Various data in other studies regarding the population’s risk of effort–reward imbalance at work are available. In a large, multicenter study of the relationship between ER imbalance and the occurrence of cardiovascular diseases, 31.7% of the study population experienced effort–reward imbalance at work [30]. In a study conducted in Germany, a representative comparison group exhibited an average ERI ratio of 0.57 [31]. Based on these data, our study assumes that employees working in the field of emergency care are more at risk with respect to effort–reward imbalance at work than are members of the general population. Taking into account the even higher exposure during the COVID-19 pandemic, the risk of an ER imbalance is likely to be even greater than it already is. Overall, very few studies have investigated the risk of effort–reward imbalance at work among emergency care workers. In a study conducted in the emergency department of a hospital in the USA, 93% of emergency room nurses reported experiencing effort–reward imbalance at work [18]. In another study that investigated a significantly greater number of subjects working in emergency rooms in China, 60% of nurses experienced effort–reward imbalance at work [19]. However, comparisons of these data are limited due to the different locations in which the studies were conducted.

In the current study, 14 (40%) nurses from the total sample reported some symptoms of burnout, and 4 (11.4%) nurses faced the risk of burnout. Representative surveys have indicated a medically diagnosed lifetime prevalence of burnout in Germany of approximately 4.2% [32]. It can therefore be assumed that the burnout risk faced by emergency nurses is noticeably greater than that faced by the general population [32]. This finding is in line with meta-analyses of international studies that have reported an increased risk of burnout among both emergency nurses and nonemergency nurses [33,34]. Studies of other professional groups that exhibit similar social skills requirements have reported somewhat lower risks of burnout among kindergarten teachers (5%) [35], music school teachers (8%) [36] and nurses (7.5%) [37].

Owing to the negative effects of burnout on the quality of patient care and the health of employees, preventive measures are necessary to reduce the risk of burnout [38,39]. As a study by Thielmann et al. [40] showed, a high level of resilience leads to less stress and a lower risk of burnout. Resilience training can therefore be recommended for emergency department staff.

This study revealed that emergency nurses who experience effort–reward imbalance at work also exhibit an increased risk of burnout. This finding is in line with the conclusions of studies of nursing staff in other countries, which have revealed a link between ERI and burnout [9]. This study revealed a positive correlation between the ERI ratio and burnout risk. Furthermore, positive and negative correlations were observed between various subscales of the ERI and MBI. In particular, the reward received, especially the job promotion, correlates with the risk of burnout. Although nursing staff were paid a one-off supplement to their salary during the pandemic, remuneration has remained almost the same. This would be a starting point for minimizing the risk of burnout through higher remuneration. Other studies on these correlations among emergency nursing staff in Germany are not yet available. The evidence in this research reported negative, moderate correlations between the “job promotion” subscale and the “esteem” subscale, and the risk of burnout is particularly important here, as this evidence can be used to develop preventive measures for employees. 

Owing to the high prevalence of burnout and gratification crises among emergency nursing staff, health promotion measures should be implemented to counteract the high levels of stress observed in the central emergency department. These measures should include both situational and behavioral prevention, especially for nursing staff who exhibit high levels of ERI. Based on the data obtained in this research, improving salary and promotion opportunities in particular represent possible burnout prevention measures. Further research is needed to investigate the influence of preventive measures on the risk of burnout and gratification crises, particularly with regard to the influence of status and recognition on the risk of burnout.

## 5. Limitations

1. Owing to the small sample size investigated in this pilot study, the results of this research cannot be generalized. It would be desirable to conduct studies in emergency departments throughout Germany, which should be designed to take into account the long-term effects. To increase the validity of this research, the sample size must be increased, and this study should continue at several locations and be verified by longitudinal studies.
2. This study has only examined the correlation between ERI and burnout. The correlations were not verified through regression analyses controlling for various socio-demographic characteristics.
3. This is a convenience sample, so it is not possible to draw conclusions about all emergency personnel.

## 6. Conclusions

Nurses in the central emergency department are at increased risk of an effort–reward imbalance compared to other professional groups.

Nurses experiencing an effort–reward imbalance at work also have an increased risk of developing burnout syndrome compared to nurses without such an imbalance.

In particular, the job promotion scale shows a negative correlation with the risk of burnout.

## Figures and Tables

**Table 1:** Sociodemographic and occupational data for the total sample and the two ERI groups divided according to risk profile.

Variable	Nonrisk Group	Risk Group	Total Sample	*p*
	Mean ± Standard Deviation			
Age [Years]	35.9 ± 12.05	34.3 ± 9.44	35.1 ± 10.7	*p_t_*_-Test_ = 0.673
Years working in the CED	6.3 ± 8.0	7.6 ± 7.6	7.0 ± 7.6	*p*_Mann-Whitney_ = 0.301
	Number (%)			
Gender	7 (36.8%) Men 12 (63.2%) Woman	4 (25%) Men 12 (75%) Woman	11 (31.4%) Men 24 (68.6%) Woman	*p*_Fisher_ = 0.493

**Table 2:** Values of the ERI scales and subscales.

ERI Scales with Subscales	Nonrisk Group	Risk Group	Total Sample	*p*_Mann-Whitney_
	Mean ± Standard Deviation [Points] Median (Min–Max) [95% Confidence Interval]			
**Effort**	9.1 ± 2.33	12.1 ± 1.57	10.5 ± 2.49	<0.001
	9 (3–13)	12 (9–14)	11 (3–14)	
	[7.98–10.23]	[11.23–12.90]	[9.64–11.30]	
**Reward**	30.7 ± 3.38	21.2 ± 4.29	26.3 ± 6.10	<0.001
	31 (23–35)	21 (13–28)	27 (13–35)	
	[29.05–32.32]	[18.90–23.48]	[24.27–28.34]	
Job promotion	12.7 ± 2.16	7.9 ± 1.93	10.5 ± 3.17	<0.001
	13 (8–15)	8 (5–13)	10 (5–15)	
	[11.64–13.73]	[6.85–8.90]	[9.39–11.50]	
Esteem	9.2 ± 1.26	6.1 ± 2.31	7.8 ± 2.35	<0.001
	10 (6–10)	6 (3–10)	8 (3–10)	
	[8.55–9.76]	[4.90–7.35]	[6.99–8.56]	
Job security	8.8 ± 1.21	7.2 ± 1.60	8.1 ± 1.62	0.002
	8 (6–10)	7 (4–10)	8 (4–10)	
	[8.26–9.43]	[6.33–8.04]	[7.54–8.62]	
**Effort–Reward ratio**	0.69 ± 0.19	1.37 ± 0.27	1.0 ± 0.41	<0.001
	0.70 (0.23–0.95)	1.43 (1.00–2.04)	0.91 (0.23–2.04)	
	[0.61–0.78]	[1.22–1.51]	[0.87–1.14]	

**Table 3:** Degrees of burnout dimensions and the total MBI score.

MBI Dimensions	Degree of Expression (Range of Points)	Nonrisk Group	Risk Group	Total Sample	*p*_χ2 bzw. Fisher exact_
Emotional exhaustion	low (≤2.00)	15 (78.9%)	4 (21.1%)	19 (54.3%)	0.002
	average (2.01–3.19)	3 (15.8%)	3 (18.8%)	6 (17.1%)	
	high (≥3.20)	1 (5.3%)	9 (56.3%)	10 (28.6%)	
Depersonalization	low (≤1.0)	10 (52.6%)	6 (37.5%)	16 (45.7%)	0.511
	average (1.01–2.19)	6 (31.6%)	5 (31.3%)	11 (31.4%)	
	high (≥2.20)	3 (15.8%)	5 (31.3%)	8 (22.9%)	
Personal accomplishment	low (≤4.0)	3 (15.8%)	2 (12.5%)	5 (14.3%)	0.950
	average (4.01–4.99)	5 (26.3%)	4 (25.0%)	9 (25.7%)	
	high (≥5.0)	11 (57.9%)	10 (62.5%)	21 (60.0%)	
					
**Risk of burnout**	no burnout (0–1.49)	13 (68.4%)	4 (25.0%)	17 (48.6%)	0.012
	some symptoms (1.5–3.49)	6 (31.6%)	8 (50.0%)	14 (40.0%)	
	serious burnout (3.5–6.0)	0 (0%)	4 (25.0%)	4 (11.4%)	

**Table 4:** Correlations between the dimensions of burnout and effort–reward imbalance.

	ERI Scales with Subscales					
**MBI dimensions**	Effort	Reward	Job promotion	Esteem	Job security	ERI ratio
Emotional exhaustion	0.468 **	−0.385 *	−0.424 *	−0.134	−0.318	0.400 *
Depersonalization	0.319	−0.390 *	−0.393 *	−0.261	−0.190	−0.320
Personal accomplishment	−0.105	0.007	0.006	0.082	−0.175	−0.053
**Risk of burnout**	0.432 **	−0.408 *	−0.462 **	−0.189	−0.217	0.384 *

* *p* < 0.05; ** *p* < 0.01.

## Data Availability

There are no plans to grant access to the full protocol, participant-level datasets, or statistical codes as data contain potentially identifying information.
